# Measuring carbonate dissolution rates under well-controlled conditions for reactive CO_2_-water flow in a large lab-scale karst fracture imitate

**DOI:** 10.1016/j.mex.2025.103271

**Published:** 2025-03-19

**Authors:** Bettina Strauch, Martin Zimmer, Kai Wendel, Leon Keim, Holger Class

**Affiliations:** a GFZ Helmholtz Centre for Geosciences, Potsdam, Germany; b Institute for Modelling Hydraulic and Environmental Systems, University of Stuttgart, Stuttgart, Germany

**Keywords:** Carbonate dissolution, CO_2_-rich water, Flow-through experiments, Lab-based simulation, large karst fracture imitate, A large lab-scale karst fracture imitate to investigate carbonate dissolution rates under well-controlled conditions

## Abstract

This study explores the carbonate dissolution dynamics in karstic systems by simulating reactive water flow under controlled large lab-scale laboratory conditions.

Using a 1 m² Jura limestone tile, the experiments focus on carbonate dissolution in fractures under variable parameters, including CO_2_ concentration (0-100 %), fluid flow velocity 50-1000 ml/l) and fracture aperture (2-10 mm). This large lab-scale setup bridges the gap between field-scale phenomena and small-scale laboratory studies.

Preliminary tests confirmed the suitability of the limestone for dynamic experiments, in terms of measurable calcium release at different experimental modifications. A novel, adjustable polyoxymethylene (POM) frame ensures precise control of flow and reaction boundaries. Process water with no CO_2_ addition, 50% CO_2_-saturation and full CO_2_-saturation were used, to gain insight into the dissolution efficiency at these CO_2_-saturation levels. The results showed, that the effects of different CO_2_ additions were well reflected in the limestone dissolution rates.

This setup provides important experimental data for the validation of numerical models for reactive transport in karst systems, to improve the understanding of the interplay between chemical reactions, fluid dynamics and geological settings.

The findings have implications for karst hydrology, geochemical modeling related subsurface processes, supporting advancements in predictive capabilities for natural and engineered systems.

## Graphical abstract

**Figure:**
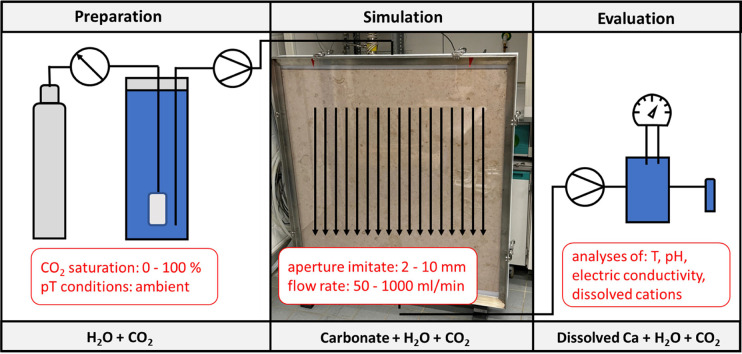

Specifications table

**Table:** 

Subject area:	Earth and Planetary Sciences
More specific subject area:	carbonate dissolution in karst systems
Name of your method:	A large lab-scale karst fracture imitate to investigate carbonate dissolution rates under well-controlled conditions
Name and reference of original method:	not applicable
Resource availability:	not applicable

## Background

About ten percent of the continental surface is covered by karstic rocks [1] with CO_2_ dissolved in water representing the main driving force of karstification and speleogenesis. Epigenic karstification occurs due to near-surface CO_2_ sources coming into contact with meteoric water. Major sources are, dependent on moisture and temperature, the metabolism of microorganisms and root respiration of plants in the uppermost soil layers [[2], [3], [4], [5]]. Relatively uniform karst denudation occurring extensively consumes most of the dissolution capacity, while still there is growth of cavities observed deep inside karstic rocks. Literature provides two widely accepted models to explain this. The first one is based on mixing corrosion, which states that the mixing of two water streams, e.g., in joints, generates calcite-aggressive behavior [6], even if both waters were before in calco-carbonic equilibrium. The second model assumes non-linear dissolution kinetics, which leave a residual carbonate dissolution potential in the meteoric water while it percolates into the rock [7,8]. A further mechanism, well-known from carbon capture and storage but so far not addressed in karst literature, has recently been proposed by Class et al. [9]. In contrast to the established models, which require water flows in conduits, this mechanism can replenish CO_2_ also in stagnant water bodies at the epiphreatic interface to air in the vadose zone. Class et al. [9] showed that dissolution of CO_2_ into stagnant water bodies is enhanced by density-driven convective fingering regimes. To be relevant for karstification, it requires episodic (quasi-)stagnant water with low CO_2_ concentrations as well as high CO_2_ concentrations in the vadose air [[10], [11], [12]]. Importantly, this preliminary work has not yet considered the full interaction of the CO_2_-water-carbonate system, i.e., the reactive flow at calcite surfaces. It is expected that dissolution of carbonates further affects density changes significantly, and thus also the convective dissolution regimes. Lacking data on the dynamics of this system on the relevant scales, mathematical-numerical modeling remains the viable option to assess the impacts of these coupled processes on karst systems.

It has been shown for different applications that joint experimental and mathematical numerical research with experiments on the laboratory scale imitating field-scale features can substantially increase process understanding, e.g., in carbon capture and storage [13,14] or in the development of biomineralization technologies [15]. In order to better understand the proposed mechanism for karstification, the validation of a reactive flow and transport model is intended to be achieved with data generated by means of a large lab-scale karstic fracture imitate of 1 m² in the lab. The generation of data sets of that real experimental simulations at well-defined and well-controlled initial and boundary conditions is necessary to close the gap between laboratory simulations and naturally occurring processes. It is the fundamental strategy of validation to study how accurate numerical simulation results compare with the physical simulation [16].

## Method details

### Materials

Deionized water, gaseous CO_2_ (AirLiquide, purity 99.98%) and a 1m² limestone surface were used to investigate the interaction of the CO_2_-water-carbonate system in the laboratory.

To realize lab-based observations of carbonate dissolution processes for a real karstic system, a massive limestone tile was used, that is as homogeneous as possible with the dimensions of 1 m x 1 m x 0.02 m with a polished surface, supplied by Fa. Franken Schotter. The limestone is a variant of Jura limestone, which is quarried from a pit near Dietfurt close to Treuchtlingen in Bavaria/Germany. With a mining area of more than 200 hectares, it is recently the largest contiguous quarry area in the Jura limestone segment. The limestone tile is overall light brownish, gray to cream-colored with inhomogeneously distributed, irregularly bounded intraclasts in a peloid matrix. Based on petrographic observations and its geological type locality, the rock is classified as “Dietfurter Limestone”. According to the classification of Dunham [17], it is a wackestone.

### Preliminary investigation on the suitability of limestone for large lab-scale experiments

The composition of the limestone, its reactivity in contact to process water and the effects on surface roughness were tested in preliminary experiments, to ensure that the carbonate dissolution experiments result in a sufficiently high ion concentration to be within the detection limit of the instruments (ICP-OES, conductivity and pH meter) planned to be employed for process water analyses.

### Limestone composition

The chemical composition of the limestone was analyzed from a dissolved limestone sample by ICP-OES. For this, a representative sample of limestone was dissolved in hydrochloric acid (10 Vol%). The result is presented in Table 1. Normalized data indicate, that nearly 99% of the dissolved cations are Ca²^+^ ions. Therefore, it is assumed that the usage of Ca2+ concentration is sufficient to conclude on dissolution behavior of the limestone tile in terms of reactivity at variable conditions in further investigations. Impurities, such as dolomitic fractions, can be neglected.

**Table 1:** Cation composition of a dissolution of “Dietfurter limestone”, b.d. indicates values below detection limit.

cations in solution	Ca	Al	Ba	Fe	K	Mg	Mn	Na	Si	Sr	Zn	sum
mg/kg	385,000	50	12	697	b.d.	3203	144	99	68	106	b.d.	389,379
wt%	98.88	0.01	0.00	0.18	–	0.82	0.04	0.03	0.02	0.03	–	100

All cation analyses were conducted using a 5110 ICP-OES with Vertical Dual View configuration (Agilent Technologies). Details can be found in the data publication of Strauch et al. [18].

### Limestone reactivity in small-scale experiments

**Fig. 1:**
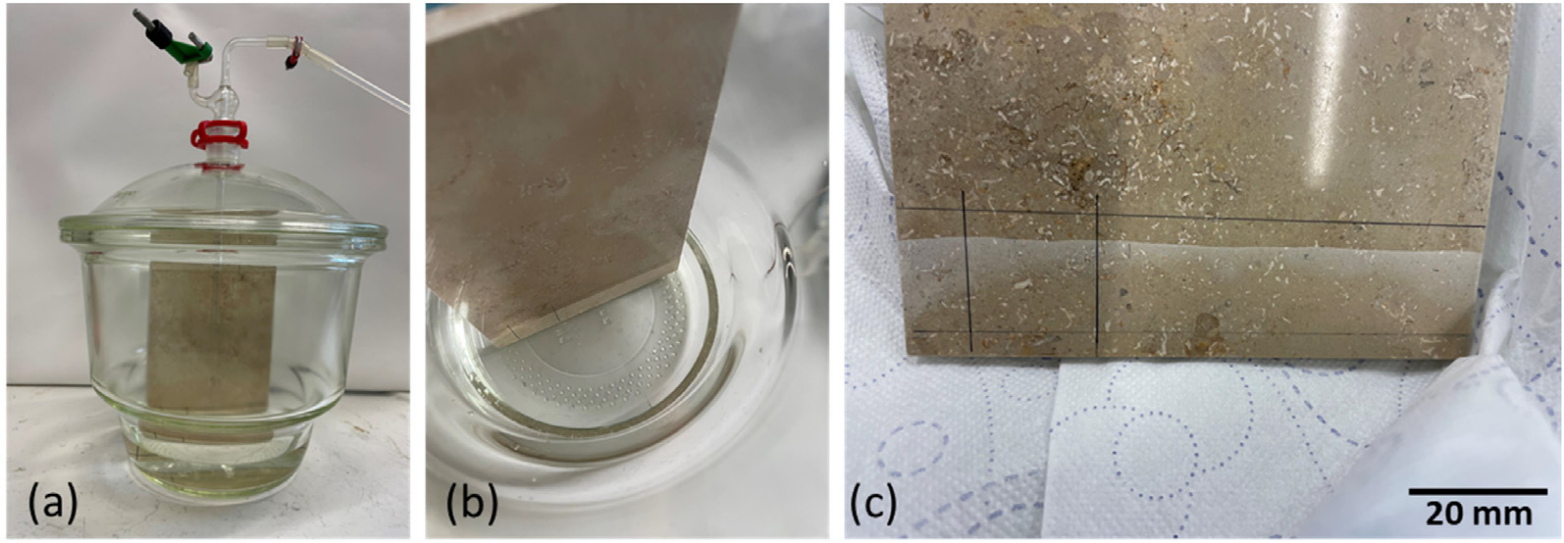
First desiccator experiments at room conditions using (a) a large desiccator with gas inlet/outlet in the lid. It holds a small limestone plate and with 365 ml of water, a liquid level of 20 mm on the plate was created (b). The surface area of the reactive limestone is 62.4 cm² (area in water). (c) After the experiment, a clear reaction edge can be seen on the tile surface.

A 120 mm x 20 mm x 9 mm tile, made of “Dietfurter Limestone”, was used for this purpose. A desiccator was equipped with the upright tile and a stagnant water column of 20 mm deionized water, corresponding to 365 ml volume, was created (Fig. 1). This resulted in a limestone surface of 62.4 cm² to be in contact with the water. The desiccator was closed and left for several days to reach equilibrium. Test 1 involved the tile, placed upright into the water, and an ambient air headspace. Test 2 was performed similarly, but with a headspace of pure CO_2_, created by flushing the system with gaseous CO_2_ for one hour. Prior to these tests, a blank experiment was carried out with a water-air situation in the desiccator, without the limestone tile. All tests were conducted at ambient temperature and pressure.

With the design of this setup, we aimed to obtain information about the solubility of the limestone tile under ambient pressure and temperature conditions as planned for the large lab-scale experiment. The resulting liquid phase was analyzed for pH, temperature, conductivity, and concentration of dissolved cations. The data are shown in Table 2; they confirm that the expected dissolved cations (mainly Ca^2+^) are within the detection limit of the ICP-OES instrument. The high Ca^2+^ concentrations in these stagnant experiments suggest, that also in a dynamic system the resulting process water of the envisaged large lab-scale experiments will be sufficiently enriched with dissolved Ca^2+^ ions.

**Table 2:** Results of the preliminary tests using a exicator for stagnant water-CO_2_-carbonate experiments.

#	Experiment	head space	duration	c_Ca_	conductivity	pH	T
			d	mg/l	µS/cm		°C
blank	water	air	7	0	0	5.81	20.9
Test 1	limestone tile in water	air	20	21	118	7.93	20.9
Test 2	limestone tile in water	CO_2_	9	332	1955	6.01	22.2

### Characterization of the limestone surface

**Fig. 2:**
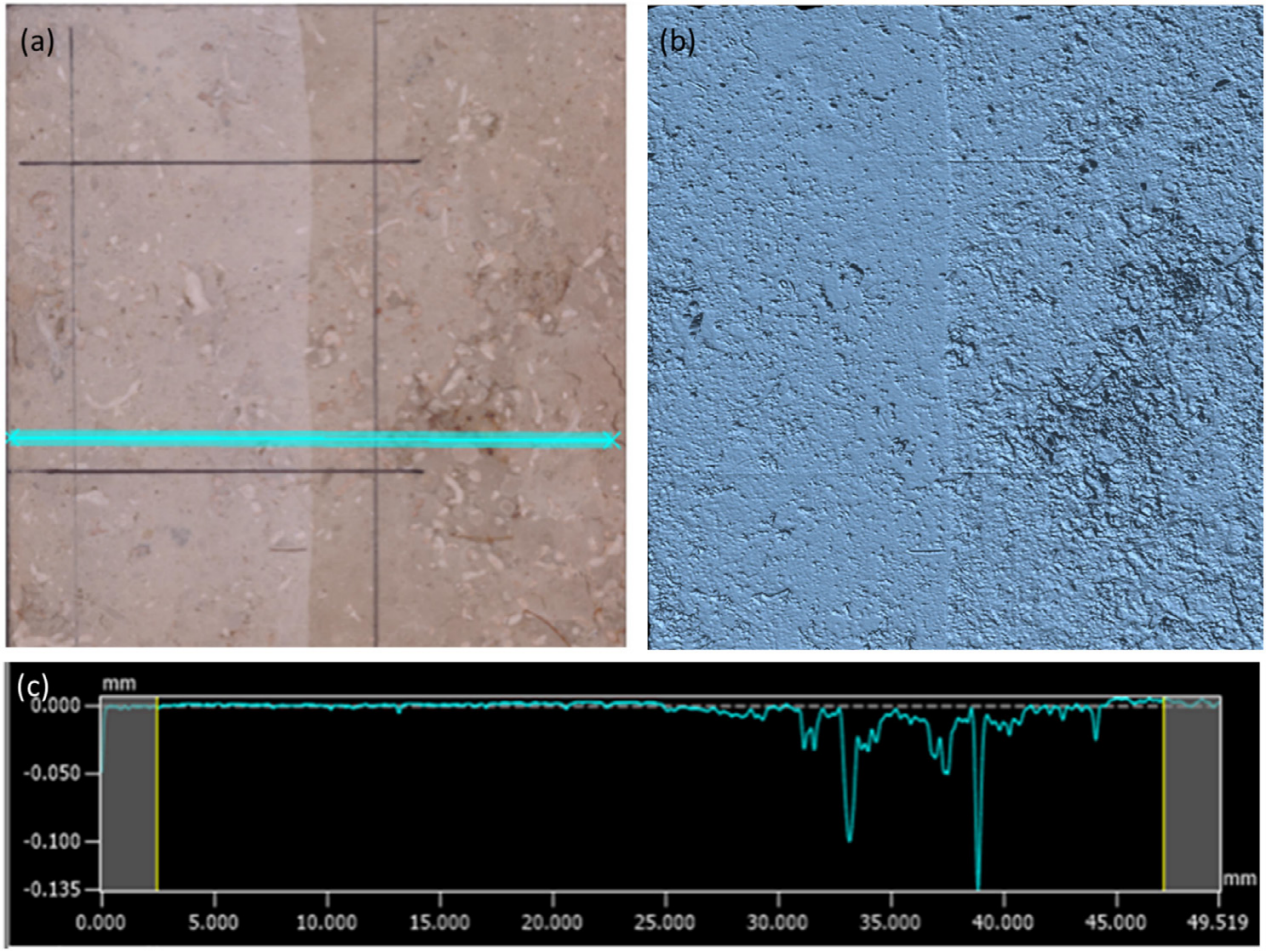
Observation of surface morphology of the limestone tile following the stagnation experiment. (a) shows a photographic image where the turquoise line marks a distance of 50 mm, (b) similar area recorded with a 3D surface profilometer (Keyence VR 3000 microscope), (c) overall profile over the length of the turquois line, showing the negative surface roughness of the original polished limestone and the uniform flattening of the surface after a stagnant dissolution experiment.

The surface of the polished limestone tile was examined using a Keyence VR 3000 microscope. Based on the investigation of several small tiles, we conclude that the pristine, polished tile has a natural negative surface roughness of up to 0.015 mm. This is attributed to inhomogeneities caused by partially mineralized fossil inclusions that break out during the polishing process due to different hardness. Fig. 2 shows a section of the small tile described in the experiment above. It shows a strong smoothing of the rock surface in terms of roughness. It is assumed that the surface in the stagnant setting is not only exposed to the strong abrasive force of the water, but also to the recrystallisation of a superficial carbonate film uniformly in the surface area. This effect should be taken into account when transferring carbonate solubility experiments from stagnant systems to dynamic systems. Otherwise, it is likely that the efficiency of the carbonate dissolution will be underestimated.

## Experimental setup

The aim was, to set up an experiment with a strong emphasis on well-defined boundary conditions for flow and carbonate dissolution while realizing a scale of relevance for karstic conditions. Further, the approach included the investigation of how different CO_2_ concentrations in the initial solution affect the development of the dynamic coupled reactive flow system. The limits of feasibility were reached with a 1 m x 1 m dimensioned limestone tile. This concerned questions of weight, the manufacturing possibilities of tile and frame, and the usability in a standard geochemical laboratory.

The rear frame was manufactured by CNC technique from a single piece of polyoxymethylene (POM). Due to its density (1.39 - 1.42 g/cm³), the polymer has sufficient hardness, strength and surface quality and is therefore suitable for the construction of parts with tight tolerances. POM is easy to machine and is characterized particularly by its high dimensional stability, which makes it an ideal material for the application here.

**Fig. 3:**
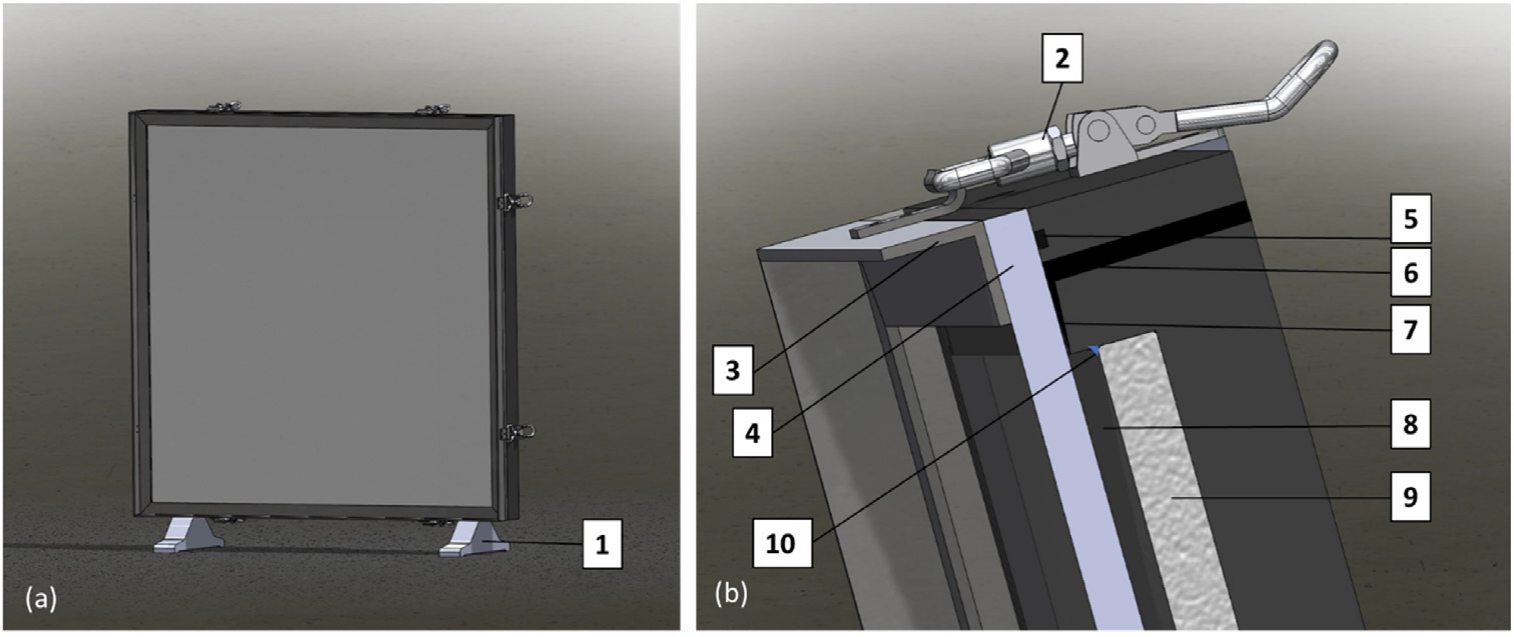
(a) Schematic drawing of the frame. (b) Sectional view of the upper part of the frame. See text for explanation. The limestone tile was emplaced into the POM frame, a macrolon© cover plate was attached and fixated by the metal frame and pressed on by a spring clip. The aperture for fluid transport was located between cover plate and limestone plate.

As shown in Fig. 3, two stands (1) were attached to the bottom side of the POM-frame in order to set up the POM frame safely secured. Two toggle fasteners (2) were attached to each side of the POM frame. The counterparts were attached to the metal frame (3). This frame was used to hold a 20 mm thick polycarbonate (macrolon©) cover plate (4) in front of the frame. A silicon tube (2.8 mm outer diameter) was used as seal between the frame and the cover plate. It was inserted into a 3 mm groove (5) that runs continuously around the POM frame. The POM frame was designed with two liquid inlet ports with a diameter of 10 mm (6) in the upper part of the POM frame. To ensure an even distribution of the fluid over the entire surface of the limestone tile, the two inlet holes were aligned with a 0.5 mm recess (7). In this way, the incoming fluid distributed along the recess before entering the aperture (8) between the tile surface and the cover plate in a homogenous flow.

The identically shaped design was located in the lower part of the POM frame and served as a fluid drain during the experiment.

The limestone tile (9) was inserted into the POM frame and bonded to the POM frame with silicone (10). A two-component silicone rubber (SILIXON10) was distributed into the small openings between frame and tile by using a pipette. The advantage of the sealing material was, that it can be easily removed without leaving stains or residuals along the limestone tile. In addition, the non-viscous consistency of the silicone has proven to be practical for a tight bondage between the POM frame and limestone tile. In this way, the flanks and back of the limestone tile were surely excluded from the carbonate corrosion in the course an experimental run.

The fluid flow itself was regulated by pumps. A KNK Neuberger pump was used on the upper entrance. The lower pump was only used for filling and flushing the system. A peristaltic pump (PA-B1, Jahnke und Kunkel, IKA Labortechnik) was used here.

The effective aperture of an experiment was adjustable prior to the assemblage of the experimental setup. By placing one or a variety of PVC spacer tiles of similar extension but in 3 mm and 2 mm thickness behind the limestone tile, the aperture could be varied between 10 mm (no PVC spacer) and 2 mm (8 mm PVC spacer).

## Preparation of process water

Each of the experimental runs was conducted with similar settings but with three different types of process-water. The water was supplied via containers (12 l volume) and taken fresh from a Millipore facility (Seralpur Pro 90CN) to ensure ion-free initial conditions (<0.05 µS/cm). This was used in the same way as for the experiments with CO_2_-free process water. When process water with 50 % CO_2_-saturation was applied for the experiment, the desired CO_2_ concentration was produced as follows. First, the water tank was filled with 6 l deionized water and flushed with gaseous CO_2_ for approximately one hour. During this time, the conductivity was measured continuously and if the values remain constant, saturation was assumed. This was usually at conductivity values of 40 µS/cm^-1^. The water tank was filled up with 6 l of CO_2_-free water and the experimental run started immediately. The water tank was kept closed during the experiment and contained a CO_2_ headspace. To produce a fully CO_2_-saturated process water, a similar procedure was applied, but the final dilution was omitted in that case.

The CO_2_ was supplied from a gas cylinder (purity 99.9%) and introduced to the water via sintered metal rod to ensure a fine gas dispersion in the water.

## Experimental procedure

Prior to each experiment, the aperture was rinsed three times with deionized water to remove any residue from previous experiments. The aperture was then filled with process water and left to stagnate for one hour.

The experimental run started by actively pumping a defined volume of process water per time unit from the 12-L storage tank through the upper two inlet tubings of the POM frame into the small recess and distributed homogenously into the aperture.

At the same time, the lower openings were used to drain the process water and collect fluid sample material for the subsequent analyses. The sampling interval depended on the flow rate of the experiment and varied from one sample every 1 l to one sample per 0.2 l of process water discharge. Immediately after sampling, the pH value, temperature, and conductivity were measured using a WTW 340i equipped with a TetraCon 325 and a SenTix 41.3 pH electrode.

The duration of the experiments varied depending on aperture and fluid flow velocity. All experimental runs were carried out until no more changes in pH value and conductivity could be observed and equilibrium was reached. Finally, the aperture volume was emptied by switching off the upper pump and allowing air to flow in, to avoid pressure drop.

**Fig. 4:**
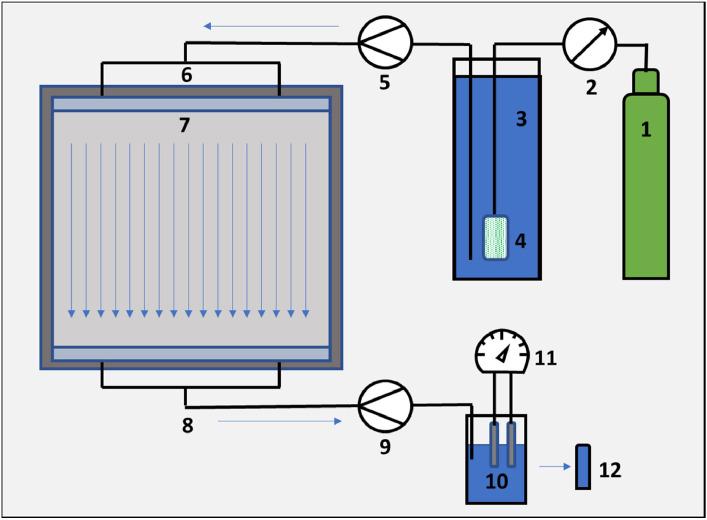
Scheme (not to scale) of the experimental setup. (1) CO_2_ gas cylinder, (2) pressure regulator for gas supply, (3) process water reservoir tank, (4) sintered metal for gas dispersion, (5) upper pump to regulate flow velocity, (6) liquid inlet through two inlets and a (7) recess, similar outlet design, (9) lower peristaltic pump, (10) process water collection beaker for (11) measurement of pH values, conductivity and temperature and to obtain (12) samples for cation analyses.

The schematic flow chart of Fig. 4 illustrates the experimental setup.

## Pre-experiments and adjustments

Before starting the experiments with the limestone tile, an inert PVC plate of identical dimensions was used to test and establish a homogenous inflow regime of process water and to practice the handling and general workflow during the experimental run.

**Fig. 5:**
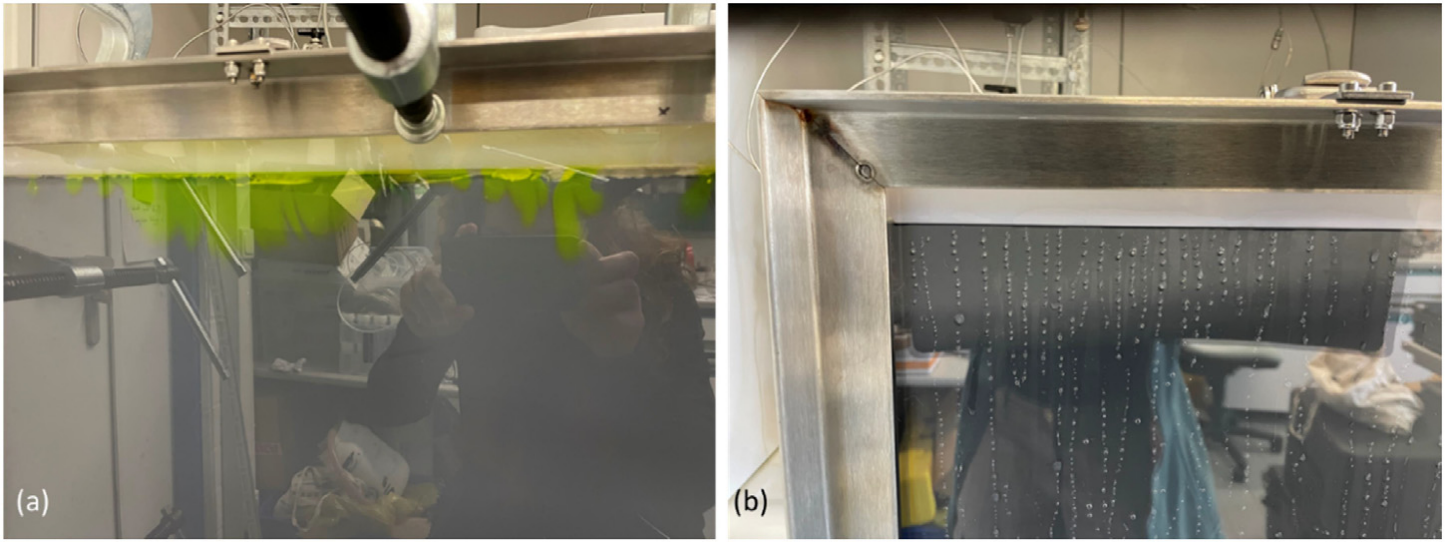
Preliminary experiments to test distribution of incoming water along the upper frame using water doped with fluorescent dye (a), or working with the aperture emptied, to observe movement, velocity and distribution of the droplets.

In order to ensure a uniform inflow of process water, several runs with fluorescent dye (Fa. Hanse Pro, disodium salt 20 mg/l) were performed and the upper recess width was corrected with additional clamps (Fig. 5a) to compensate for small irregularities due to water pressure changes causing slight bulging of the cover plate. Also, slowly-dripping water into a partially filled aperture was employed to visualize the process water entry behavior in terms of distribution and rate (Fig. 5b).

**Fig. 6:**
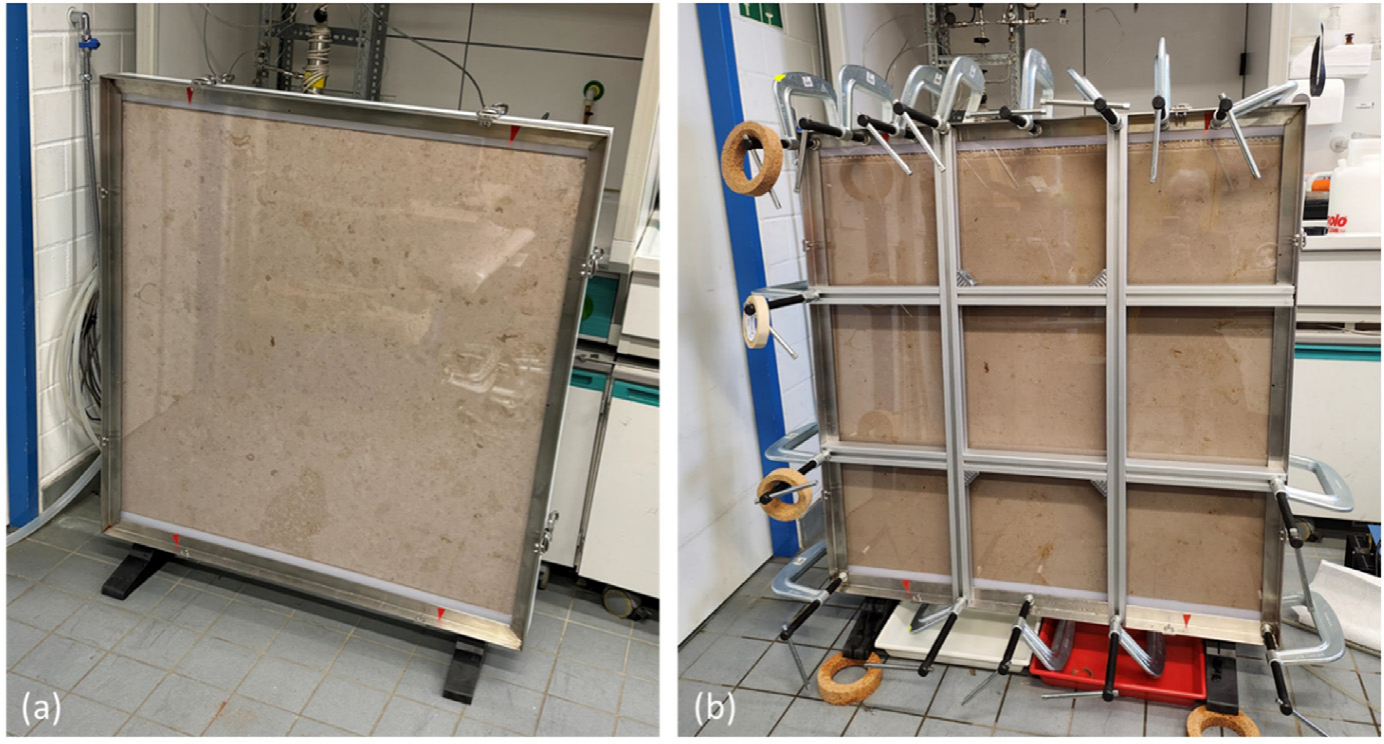
(a) Original experimental setup in the laboratory. (b) Modifications required due to the bulging cover plate and the adjustment of the inflowing process water to ensure homogenous distribution across the entire aperture width.

During the preliminary tests it was found, that the polycarbonate cover plate bulged outward due to increasing water pressure in the aperture. To avoid this effect, an additional grid frame (Fig. 6b) had to be applied to the setup (Fig. 6a), as the accuracy of the aperture width is of great importance to assure the well-controlled conditions needed for precise data acquisition as prerequisite for the subsequent modelling approach.

### Method validation

**Fig. 7:**
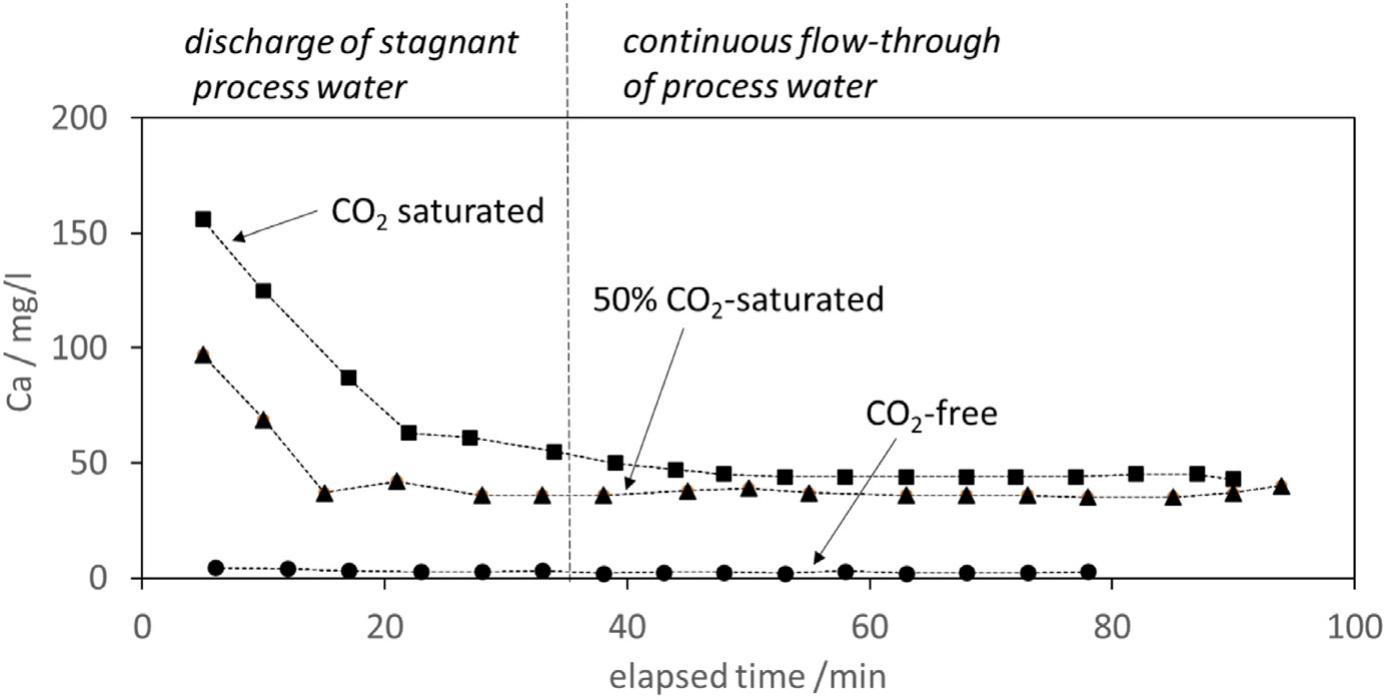
The Ca^2+^ concentration in the discharged process water in the course of three experiments using a 6 mm aperture and three different CO_2_ concentrations in the initial process water. It is divided into the initial phase of stagnant process water discharge and the continuous flow-through phase.

Fig. 7 illustrates the experimental runs by plotting exemplarily the Ca^2+^ concentration of the released process water of three laboratory simulations with varying CO_2_ content in the initial process water. The series was performed with an aperture width of 6 mm and a fluid flow rate of 200 ml/min. Process water samples were taken approximately every 5 minutes.

The experimental run started by loading 6 l of process water into the aperture via pump from bottom up, to allow the release of air from the aperture through the upper openings. Then, the process water remained stagnant in the aperture for 1 hour before the dynamic phase of the experiment started.

Now, process water was added through the top openings and the fluid flow rate was set to 200 ml/min. When 1 l water was released from the system, the first sample was taken. The gained sample was used for immediate pH, temperature, and conductivity measurements and subsequent Ca^2+^ analyses. All data are available in the data publication of Strauch et al. [18]. When the next liter of process water drained from the system, the next sample was taken and analyzed. The procedure continued until no more changes in conductivity measurements were observed. The changes in Ca^2+^ concentration over time are shown in Fig. 7 for experiments with CO_2_-free, partly saturated and fully saturated process water, respectively.

## Observations

The dissolution process in all setups demonstrated a typical behavior, which can be subdivided in two steps:

*(1) Initial phase of discharging stagnant process water*

Within the first approximately 35 minutes of the experimental run, all of the stagnant process water was discharged, resulting in decreasing Ca^2+^ concentrations in the outflow sample. Naturally, the gradient of Ca^2+^ concentration decrease is stronger in CO_2_-rich process water, as here the initial concentration of Ca^2+^ is highest. The stagnant phase resulted in approximately 5, 100 and 160 mg/l Ca^2+^ in the initially CO_2_-free, partly saturated and saturated process water, respectively.

*(2) Continuous flow-through phase with plateau*

In all experiments, the initial phase, with decreasing ion concentrations, was followed by a phase of constantly in and out flowing process water where the Ca^2+^ concentration remained fairly constant throughout time (approximately one hour). This indicated that equilibrium conditions were established. As to expect, the level of equilibrium in terms of Ca^2+^ dissolution differs, with lowest ion freight observed in CO_2_-free process water and highest ion freight in CO_2_-saturated process water. For the set of experiments shown here, the concentrations varied from less than 5 mg/l Ca^2+^ in CO_2_-free process water to 50 mg/l Ca^2+^ in the CO_2_-saturated process water. When the system approached equilibrium, reflected by observation of stabilizing conductivity values during the experimental run, the inflow of process water was stopped.

## Relationship between CO_2_-saturation and carbonate dissolution

The results highlight the significant role of CO_2_-saturation in enhancing carbonate dissolution through acidification, as observed through variations in conductivity, Ca^2+^ concentration and pH in the three experimental runs shown here.

*CO_2_-free process water:* The minimal changes in conductivity and Ca^2+^ concentration confirmed the limited dissolution of carbonate in neutral pH conditions. The stable pH (8.0 -8.5) indicates a buffering effect typical of carbonate systems in CO₂-absent environments. This setup may serve as a control, demonstrating baseline dissolution rates under neutral conditions.

*50% CO_2_-saturated process water*: The initial low pH (3.8) indicates the acidifying effect of CO_2_. Over time, the pH stabilizes (∼5.9), indicating progressive neutralization as dissolved carbonate ions buffer the system. The significant rise in Ca^2+^ concentration (up to 110 mg/l) and conductivity underscore the enhanced dissolution rates compared to CO_2_-free water. This suggests partial CO_2_ saturation is sufficient to drive carbonate dissolution, albeit less efficiently than fully saturated conditions.

*100% CO_2_-saturated process water:* The sharpest initial drop in pH (∼3.6) reflects the highest acid concentration and the strongest dissolution rate during the initial stagnant phase. Over time, the pH increased slightly (∼5.8), but the system remains highly acidic, promoting continuous dissolution. The conductivity values and Ca^2+^ concentrations were significantly higher than in previous experiments, with Ca^2+^ concentrations up to 179 mg/l which suggests very high dissolution of the available carbonate material.

The results show, that CO_2_ addition increases the carbonate dissolution in the order of a magnitude. All data can be found in the data publication of Strauch et. al. [18]. A detailed discussion of the results, in comparison with results of numerical models will be carried out in the corresponding publication of Wendel et al. [19].

## Conclusions

The experimental setup presented here offers a robust method for the investigation of reactive transport phenomena in carbonate systems. The validated methodology allows for improved data-based numerical modeling to simulate karst system evolutions with greater accuracy and confidence.

The novel approach of well-defined large laboratory-based experiments contribute to a deeper understanding of karst dynamics. Overall, it emphasizes the importance of integrating experimental and modeling approaches to tackle challenges in geological carbon systems.

## Limitations

The setup is limited to experiments at ambient pressure and temperature conditions. For temperature, variations can be made by placing the entire setup into a climatised room.

## Ethics statements

not applicable

## Declaration of competing interest

The authors declare that they have no known competing financial interests or personal relationships that could have appeared to influence the work reported in this paper.

## Data Availability

The complete data set is availbable in the data publication of Strauch et al. [18], see references.
